# The US *SimSmoke* tobacco control policy model of smokeless tobacco and cigarette use

**DOI:** 10.1186/s12889-018-5597-0

**Published:** 2018-06-05

**Authors:** David T. Levy, Zhe Yuan, Yameng Li

**Affiliations:** Lombardi Comprehensive Cancer Center, Georgetown University, 3300 Whitehaven St., Suite 4100, Washington DC, USA

**Keywords:** Smokeless tobacco, Tobacco control policies, Simulation model

## Abstract

**Background:**

Smokeless tobacco (SLT) prevalence had been declining in the US prior to 2002 but has since increased. Knowledge about the impact of tobacco control policies on SLT and cigarette use is limited. This study examines the interrelationship between policies, cigarette use, and SLT use by applying the *SimSmoke* tobacco control policy simulation model.

**Methods:**

Using data from large-scale Tobacco Use Supplement and information on policies implemented, US *SimSmoke* was updated and extended to incorporate SLT use. The model distinguishes between exclusive SLT and dual use of SLT and cigarettes, and considers the effect of implementing individual and combined tobacco control policies on smoking and SLT use, and on deaths attributable to their use. After validating against Tobacco Use Supplement (TUS) survey data through 2015, the model was used to estimate the impact of policies implemented between 1993 and 2017.

**Results:**

*SimSmoke* reflected trends in exclusive cigarette use from the TUS, but over-estimated the reductions, especially among 18–24 year olds, until 2002 and under-estimated the reductions from 2011 to 2015. By 2015, *SimSmoke* projections of exclusive SLT and dual use were close to TUS estimates, but under-estimated reductions in both from 1993 to 2002 and failed to estimate the growth in male exclusive SLT use, especially among 18–24 year olds, from 2011 to 2015. *SimSmoke* projects that policies implemented between 1993 and 2017 reduced exclusive cigarette use by about 35%, dual use by 32.5% and SLT use by 16.5%, yielding a reduction of 7.5 million tobacco-attributable deaths by 2067. The largest reductions were attributed to tax increases.

**Conclusions:**

Our results indicate that cigarette-oriented policies may be effective in also reducing the use of other tobacco products. However, further information is needed on the effect of tobacco control policies on exclusive and dual SLT use and the role of industry.

## Background

Adult smoking prevalence in the US declined from 26% in 1993 to 14% in 2015 [1]. Much of that decrease can be attributed to the implementation of tobacco control policies, including smoke-free air laws, marketing restrictions, media campaigns, treatment and tax increases [2, 3]. While smoking prevalence has declined, the use of other tobacco products, such as little cigars or smokeless tobacco (SLT), and of e-cigarettes has increased [4–7]. Much of that is multi-product use, of which 60% includes cigarettes [7].

Although male SLT use had declined in the US from 4.2% in 1993 to 2.8% in 2002 [8, 9], it increased to 3.0% by 2011 [6, 10, 11], with snuff sales increased by 65% [12]. SLT use has been shown to be a direct cause of oral and esophageal cancer, and may also cause heart disease, gum disease and oral lesions [13]. With concerns about the health effects and increasing use of SLT, some states have directed policies at reducing SLT use, including increased SLT taxes, educational campaigns, and cessation treatment [14, 15]. In addition, the 2009 Family Smoking Prevention and Tobacco Control Act (FSPTCA) authorized the Food and Drug Administration to regulate the marketing, promotion and sale of cigarettes and SLT.

Policies directed at reducing SLT use may also impact cigarette use. For example, cigarette use may increase if youth and young adults initiate smoking instead of SLT or if smokers are discouraged from using SLT to help quit cigarette use. However, SLT-oriented policies could reduce cigarette use if the two tend to be used together (i.e. dual use) and the policies encourage cessation, or if SLT acts as a gateway to cigarette smoking. Similarly, policies directed at reducing cigarette use may discourage SLT use if the two are used together or may encourage SLT use if SLT is used as a cigarette substitute. Policy evaluations have provided limited information on their effects [15]. Knowledge of the policy impacts can help to better design policies towards SLT use, and may have implications for other nicotine delivery products, such as e-cigarettes [16].

This paper employs simulation modeling to examine the inter-relationship of tobacco control policies and patterns of cigarette and SLT use. We adopt the well-established *SimSmoke* simulation model [2, 3]. The model incorporates population and smoking dynamics and focuses on the major cigarette-oriented tobacco control policies, including taxes, smoke-free air laws, media campaigns, marketing restrictions, cessation treatment policies and youth access enforcement. *SimSmoke* has been used for advocacy and planning purposes to examine the impact of past and projected future policies individually and in combination [17]. The model has been developed and validated for over 25 nations and 8 states with a wide range of different policy changes [2, 18–26].

The *SimSmoke* model is extended here to incorporate SLT use, distinguishing between exclusive SLT and dual (both cigarette and SLT) use. We consider the effect of tobacco control policies implemented between 1993 and 2017 on cigarette and SLT use and on the deaths attributed to that use.

## Methods

**Fig. 1:**
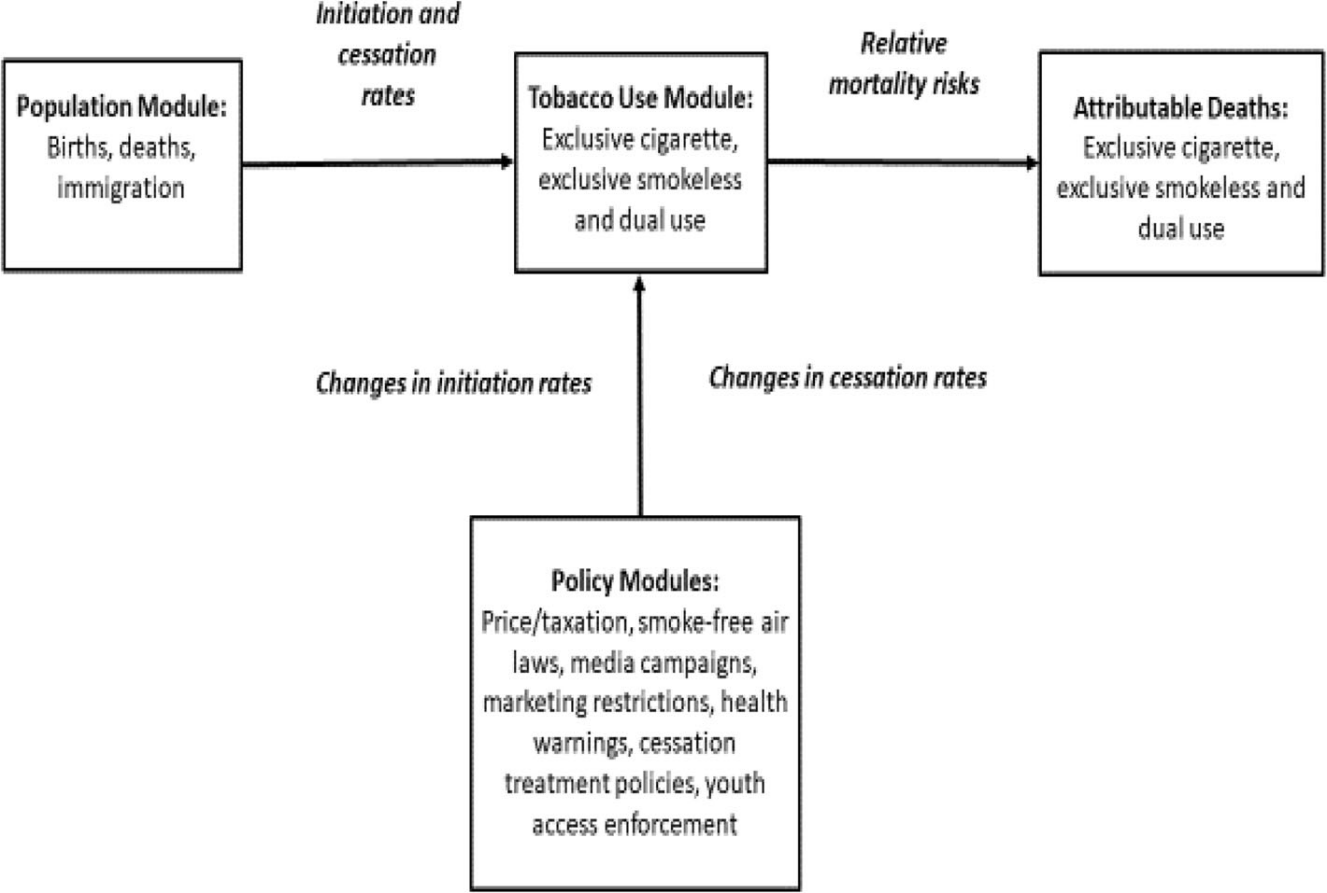
Components of the SimSmoke Smokeless Model

The model begins with the 1993 population distinguished by age and gender and further distinguished as never tobacco users, and both current and former users among exclusive cigarette, exclusive SLT, and dual users. As shown in Fig. 1, cigarette and SLT use age change over time through modules for population, tobacco use, tobacco-attributable deaths and separate modules for each policy.

### Population

Population data were obtained by single age (0 through 85) from the Census Bureau for 1993–2013 [27–29] and for 2016–2067 [30] from the Census Bureau’s Population Projections Program. Starting with the population in 1993, the population evolves through births, deaths and net immigration, with births up to age 14 based on the obtained population data and older age groups subject mortality rates from the CDC [31]. Mortality rates by age and gender were averaged by age group over the years 1999 through 2013 and then smoothed using 3-year (ages 0–3), 5-year (ages 4–24), and 10-year (ages 25–80) moving averages and extrapolated to age 85. Population predictions were adjusted by net migration rates (2014–2020 average) from the Census Bureau [32], and calibrated to Census projections.

### Tobacco use

Individuals evolve from never tobacco users to current tobacco users through smoking and SLT initiation. Tobacco users become former users through quit rates, but may return to their prior tobacco use state through relapse. A discrete time, first order Markov process was assumed for these transitions.

Baseline estimates of exclusive smoking, exclusive SLT and dual use status by age and gender were obtained from the nationally-representative 1992/3 Tobacco Use Supplement (TUS) of the Current Population Survey [33]. Current smokers were defined as individuals who have smoked more than 100 cigarettes in their lifetime and currently smoke cigarettes either daily or on some days^.^ A question was asked regarding whether the individual “regularly” used SLT. Those regular SLT users were further distinguished as dual users (with cigarette use) and exclusive SLT users. Former users were defined as those who met the respective definitions for use, but reported no current use. Former smokers were split into exclusive smokers and former dual users using the age-specific ratio of exclusive smokers and dual users, and former exclusive SLT users were estimated by the ratio of former to current smokers. Former exclusive smokers and dual users were distinguished by years since quitting (< 1, 1, 2 …, 15, > 15 years). Since former SLT users were not asked about years since quitting, the initial percentages were assumed the same as for former smokers.

Because evidence on initiation and early transitions to SLT use from the literature was mixed [34–38] and because the TUS did not provide such information, we employed a measure of net initiation, whereby initiation was measured for each of the three user groups as the difference between the base year prevalence at a given age and base year prevalence at the previous age. Thereby, this measure incorporates initiation, cessation and switching between tobacco products, similar to previous *SimSmoke* models without the ability to switch products [2, 3]. This method ensures stability and internal consistency of the model. We allowed for initiation through age 30 for males and age 27 for females, the respective ages when net initiation for all three user groups began to decline. Cessation occurs after the last age of net initiation.

Data on smoker quit rates were obtained from the TUS, measured as those who quit in the last year, but not the last 3 months [39]. Since sufficient data to estimate quit rates for exclusive SLT and dual users were not available from the TUS, we considered previous literature. Studies [40–42] generally found that quit rates were at least as high among SLT as cigarette users. With some exceptions [43], studies obtained similar quit rates for dual users and exclusive smokers [42, 44, 45]. Quit rates were set the same for dual and exclusive SLT users as for all smokers. Age- and gender-specific relapse rates by years quit were based on the rates for smokers [46–49]. Finally, since studies indicated limited switching between SLT and cigarettes, except at younger ages [40–42], switching only occurred through net initiation.

### Tobacco-attributable deaths

Relative risk estimates for current and former smokers by age and gender were based on the Cancer Prevention Study II [48, 50, 51], as in previous US *SimSmoke* models [2, 3]. Relative risks for dual users may be less than for exclusive smokers due to reduced quantity smoked [43], but studies have found similar risks [52, 53] except with large quantity reductions [54]. We assigned the same risks to exclusive cigarette and dual users, so that risks decline at the same rate with years since quitting [48, 50, 51]. We estimate an exclusive SLT relative mortality risk of 1.15 based on a large-scale US study [55].

To obtain smoking-attributable deaths, the number of exclusive smokers at each age is multiplied by the excess mortality risks (exclusive smokers death rate minus never smokers death rate) to obtain attributable deaths by age, and then summed over ages. The same procedure was applied to former exclusive smokers and summed over current and former smokers. Separate estimates were derived in the same way for exclusive SLT and for dual users.

### Policies

The model was initialized with 1993 policy levels, and incorporates US and state policy changes occurring between 1993 and 2017. Policy descriptions and effect sizes are shown in Table 1. Policies are generally modelled as having immediate effects on prevalence rates and ongoing effects through initiation and cessation rates. When more than one policy is in effect, the effects are multiplicatively applied as percent changes, subject to synergies (e.g., through publicity from media campaigns, see Table 1).

**Table 1:** Policy Inputs for Cigarette and Smokeless Tobacco in *SimSmoke* Simulation Model

Policy	Description	Cigarette Effect Size^a^	Smokeless Tobacco Effect Size^b^
Tax Policies [56, 67, 98, 99]			
Cigarette price/tax		Elasticities^c^	
	The effect of taxed is directly incorporated through average US price (including generics), with separate prices for cigarette and SLT. The price elasticity is used to convert the % price changes into effect sizes. The dual price is computed as 4/5 of the cigarette price + 1/5 SLT price	−0.4 ages 10–17	Same
		− 0.3 ages 18–24	Same
		− 0.2 ages 25–34	Same
		− 0.1 ages 35–64	Double
		− 0.2 ages 65	Same
Smoke-Free Air Policies [62]			
Worksite smoking ban	Ban in all indoor worksites, with strong public acceptance and enforcement of laws (reduced by 1/3 if allowed in ventilated areas and by 2/3 if allowed in common areas)	−6%	One-fourth
Restaurant smoking ban	Ban in all indoor restaurants (reduced by half if partial)	−2%	One-fourth
Bars smoking ban	Ban in all indoor (reduced by half if partial)	−1%	One-fourth
Other place bans	Ban in 3 out of 4 government buildings, retail stores, public transportation, and elevators	−1%	One-fourth
Enforcement	Government agency enforces the laws	Effects reduced 50% absent enforcement	Same
Mass Media Campaigns [65]			
High publicity media campaign	Campaign publicized heavily on TV and at least some other media, with a social marketing approach	−6.5%	Half
Moderate publicity media campaign	Campaign publicized sporadically on TV and at least some other media	−3.25%	Half
Low publicity media campaign	Campaign publicized only sporadically in newspaper, billboard, or some other media	−1.625%	Half
Marketing Restrictions [67, 68]			
Comprehensive marketing ban	Ban is applied to television, radio, print, billboard, in-store displays, sponsorships and free samples (all indirect marketing)	−5% prevalence,-8% initiation,+ 4% cessation	Same
Moderate advertising ban	Ban is applied to all media (television, radio, print, billboard) plus one indirect marketing medium	−3% prevalence,-4% initiation,+ 2% cessation	Same
Weak advertising ban	Ban is applied to some television, radio, print, and billboard	−1% prevalence and initiation only	Same
Enforcement	Government agency enforces the laws	Effects reduced 50% absent enforcement	Same
Health Warnings [69]			
Strong	Labels are large, bold and graphic, and cover at least 30% of pack	−4% prevalence, −2% initiation, + 10% cessation	Same
Moderate	Laws cover 1/3 of package, not bold or graphic	−2% prevalence & initiation, + 2% cessation	Same
Weak	Laws cover less than 1/3 of package, not bold or graphic	− 1% prevalence & initiation, + 2% cessation	Same
Cessation Treatment Policies [70]			
Availability of pharmacotherapies	Legality of nicotine replacement therapy, Wellbutrin and varenecline	−1% prevalence, + 6% cessation^d^	Half
Proactive quitline	A proactive quitline with publicity throughout the media campaign with no cost nicotine replacement therapy	−1% prevalence, + 8% cessation^d^	Half
Subsidization pharmacotherapy	Payments to cover pharmacotherapy and behavioral cessation treatment	−2.25% prevalence, + 12% cessation^d^	Half
Brief health care provider interventions	Advice by health care provider to quit and methods provided	−1% prevalence, + 8% cessation^d^	Half
All of the above	Complete availability and reimbursement of pharmaco- and behavioral treatments, quitlines, and brief interventions	−5.2% prevalence, + 43% cessation^d^	Half
Youth Access Restrictions [75]			
Strongly enforced	Compliance checks are conducted 4 times per year per outlet, penalties are potent and enforced with heavy publicity	−16% initiation and prevalence for ages 16–17 and − 24% ages < 16^d^	Half
Well enforced	Compliance checks are conducted regularly, penalties are potent, and publicity and merchant training are included	− 8% initiation and prevalence for ages 16–17 and − 12% ages < 16^d^	Half
Low enforcement	Compliance checks are conducted sporadically, penalties are weak	−2% initiation and prevalence for ages 16–17 and − 3% ages < 16^d^	Half
Vending machine restrictions	Total ban	Enforcement effects increase by 8%^d^	Half
Self-service restrictions	Total ban	Enforcement effects increase by 4%^d^	Half
Publicity	Media campaigns directed at youth use	Enforcement effects increase by 10%^d^	Half

^a^ Unless otherwise indicated, the effects are in terms of the reduction in prevalence during the first year, the reduction in initiation, and increase in first year quit rates during the years that the policy is in effect

^b^ Effect sizes are relative to cigarette effect sizes and applied to exclusive cigarette use only unless otherwise indicated

^c^ Elasticities translate into effect sizes through percentage change in price

^d^ Effect size differs for exclusive SLT and for dual use

In the tax module [56], prices were modeled as having constant proportional effects (i.e., constant price elasticities) with respect to price, as derived from demand studies. Based on previous reviews [56, 57], the model assigns a prevalence elasticity for exclusive cigarette and dual use of − 0.4 through age 17; − 0.3 for ages 18 to 24; − 0.2 for ages 25 to 34; − 0.1 for ages 35 to 64; and − 0.2 for age 65 and older. Price elasticities for adult SLT use have generally ranged from − 0.2 to − 0.8 [15]. The price prevalence elasticities for exclusive SLT use were estimated at − 0.4 for those through age 17, − 0.3 for ages 18–24, and − 0.2 for ages 25 and above. Cigarette prices were measured by national average cigarette retail prices (including generics) [58] for 1993–2014 with the 2014 price adjusted upward for 2015–2017 to reflect state level tax increases as weighted by the state population. The national average retail prices and manufacturer tax for SLT products through 2014 were measured by the state retail prices and manufacturer taxes weighted by the SLT smoker population [59], using manufacturer sales and quantity shipped in pounds [60], tax data [61], estimated weights per unit [60], and estimated mark-ups. We adjusted the 2014 price upward for 2015–2017 by the state population-weighted tax increase. For SLT users, we used a weighted price, with weights of 80% of the cigarette price and 20% of SLT price [59]. All prices were deflated by the consumer price index to adjust for price inflation.

*SimSmoke* considers worksites, restaurants, pub and bars, and other public places laws, and the role of enforcement [62]. Studies of SLT use have found a negative relationship to smoke-free air laws [15]. Based on these findings and since smoke-free air laws are not explicitly directed at SLT use, exclusive SLT and dual use effect sizes were set at 25% those of cigarettes. Data on state level smoke-free air laws [63] were weighted by state smoker populations. The enforcement level was set at 80% for all years, as previously developed for US SimSmoke [2, 3].

*SimSmoke* evaluates media campaigns in terms of overall tobacco control expenditures, much of which are for media campaigns [64]. They are categorized as high, medium, or low levels [65]. Studies have generally found SLT-oriented educational campaigns effective in reducing youth and adults and adult use [15], but due to reduced emphasis on SLT as compared to cigarette-oriented campaigns, exclusive SLT and dual effect sizes were set at 50% that of cigarettes. State per capita expenditures [66] were categorized by levels and weighted by the state smoker population, and were initially categorized as low level in 1993 increasing to medium level by 2004.

*SimSmoke* considers restrictions on both direct and indirect marketing [67, 68]. While no studies have directly examined the relationship of marketing restrictions to SLT use, awareness of and exposure to SLT advertisements has been associated with increased use [15]. SLT and dual use were assigned the same policy effect sizes as for cigarettes. Restrictions on advertising for both SLT and cigarette use were set at a minimal level from 1993 to 2009, reflecting an earlier media advertising ban, with enforcement set at 90% [2]. In 2010, they were increased to 25% moderate and 75% minimal, reflecting added 2009 FSPTCA restrictions on sponsorships and coupons, and in publications.

The effectiveness of health warnings depends primarily on their size and whether they include graphics [69]. Limited effectiveness has been found for text-only warnings on SLT packages, but pictorial warnings were associated with less susceptibility to SLT use among youth and greater interest in cessation among adults [15]. We assume the same effect of SLT warnings on exclusive SLT and dual use as for cigarette warnings on cigarette use. Health warnings for cigarettes have been minimal since 1966. However, since 2010, SLT packaging is required to display large text warnings covering at least 30% of two principal sides of the package, larger than cigarette warnings. SLT warnings were assigned a minimal level until 2009 and a moderate level since 2010.

Cessation treatment policy includes brief interventions, pharmacotherapy availability, financial coverage of treatments, and quitlines. [70] Reviews of randomized trials of pharmacological SLT interventions found mixed effects [13, 71, 72] and have also found behavioral interventions to promote quitting among SLT users [15]. However, SLT users currently use these resources at low rates [73]. Compared to exclusive smokers, cessation treatment policies were assigned 50% the effect on SLT users, but 100% of the effect on dual users. The levels of cessation treatment use were based on previous versions of US *SimSmoke* [2, 3, 70]. Treatment coverage was initiated in stages beginning with minimal in 1997 increasing to moderate by 2007 [74]. A national (active) quitline was implemented at 25% capacity beginning in 2003 increasing in stages to 100% by 2007 [74]. Brief interventions were set at a level of 50% for all years. Most states currently have provisions for SLT advice and treatment, and consequently the policy levels were set the same as for cigarettes.

Youth access enforcement include enforcement, and restrictions on vending machines and self-service. Strongly enforced and publicized youth access laws yield a larger reduction in youth smoking initiation for 10–15 year-olds than for 16–17 year-olds, further enhanced by vending machine and self-service bans [75]. Two studies of youth SLT use [76, 77] found youth access policies affected SLT use, although the effect was weak, and two studies [78, 79] found lower compliance rates for SLT than cigarette purchases. Youth access policy effect sizes for exclusive SLT use were assigned 50% of the effect sizes for cigarettes, while the effects on dual use were assigned the same effect sizes as for exclusive cigarette use. Enforcement levels for both SLT and cigarettes were set at none before 1997, at low-level from 1998 to 2002 and at mid-level since 2003 [6]. Levels for vending machine bans were set at 50% beginning in 1993 [80] increasing to 75% by 2000, and for self-service bans were set at 50% beginning in 1995. Both vending machine and self-service bans were increased to 100% in 2010, reflecting requirements under the 2009 FSPTCA.

### Validation

To validate the model, we compared predicted cigarette and SLT prevalence rates (that incorporate policy changes) to the comparable use rates estimated from the 2002, 2010/11 and 2014/15 TUS surveys. Because screening questions on SLT use in the TUS changed from “regular use” to days use, current users from 2002 onward were defined as individuals currently using SLT at least 10 days in the last month [81]. For the years 2002, 2010/11 and 2014/15, we considered whether *SimSmoke* predictions were within the 95% confidence intervals (CI) from the TUS, assuming a binomial distribution for each use category. We also compared the relative change in prevalence rates from *SimSmoke* to those from the TUS by sub-periods (1993–2002, 2002–2011, and 2011–2015) and overall (1993–2015).

### The effect of past tobacco control policies

Upon validating the model, we estimated the effect of policies on tobacco prevalence and tobacco-attributable deaths. First, we programmed *SimSmoke* with all policies remaining at their 1993 levels to estimate the counterfactual without any policies implemented. We then subtracted estimates incorporating all implemented policies from those for the counterfactual in order to estimate the net reductions due to the policies implemented since 1993. The contribution of individual policies were estimated by reprogramming *SimSmoke* to only allow for the change in that policy while holding other policies constant, which was compared to the counterfactual with no policies implemented. The relative reductions for each policy were measured relative to the summed effects of all policies, since the effects with multiple policies depend on assumed synergies and do not sum to one.

## Results

### Predictions of smoking and SLT prevalence from 1993 to 2014/15

*SimSmoke* predictions for 1993 to 2015 incorporating policy changes and estimated smoking prevalence from TUS are shown for exclusive cigarette, dual and exclusive SLT users in Table 2.

**Table 2:** Validation: Exclusive Cigarette, Dual and Exclusive SLT Use, *SimSmoke* Projections vs.Tobacco Use Supplement, by Age and Gender, 1993–2015

EXCLUSIVE CIGARETTE USE									
Ages	Source	1993	2002	Relative change^a^1993–2002	2011	Relative change^a^2002–2011	2015	Relative change^a^2011–2015	Relative change^a^1993–2015
Male									
18+	SimSmoke	25.6%	20.2%	−21.3%	15.4%	−23.5%	14.2%	−8.2%	−44.7%
	CPS-TUS	25.7%	22.0%	−14.1%	17.1%	−22.5%	14.9%	−12.6%	−41.8%
	95% CI		(21.7, 22.4%)		(16.8, 17.4%)		(14.7, 15.2%)		
18–24	SimSmoke	25.1%	20.4%	−18.7%	17.0%	−16.4%	16.9%	−1.0%	−32.8%
	CPS-TUS	25.5%	26.8%	5.4%	18.7%	−30.2%	15.6%	−16.7%	−38.7%
	95% CI		(25.7, 28.0%)		(17.8, 19.7%)		(14.6, 16.6%)		
25–34	SimSmoke	29.0%	23.9%	−17.6%	20.2%	−15.4%	19.3%	−4.4%	−33.3%
	CPS-TUS	29.0%	24.2%	−16.6%	21.2%	−12.5%	18.0%	−15.2%	−38.1%
	95% CI		(23.4, 25.0%)		(20.5, 21.9%)		(17.3, 18.7%)		
35–54	SimSmoke	29.5%	22.0%	−25.2%	15.7%	−28.8%	14.1%	−10.1%	−52.1%
	CPS-TUS	29.6%	25.5%	−13.8%	19.2%	−24.9%	16.7%	−12.9%	−43.6%
	95% CI		(24.9, 26.1%)		(18.7, 19.6%)		(16.2, 17.2%)		
55+	SimSmoke	17.4%	14.9%	−14.4%	11.7%	−21.0%	10.4%	−11.5%	−40.1%
	CPS-TUS	17.5%	14.5%	−17.0%	12.7%	−12.4%	12.2%	−4.2%	−30.3%
	95% CI		(14.0, 15.0%)		(12.3, 13.1%)		(11.8, 12.6%)		
Female									
18+	SimSmoke	22.1%	17.3%	−21.4%	13.4%	−22.5%	12.4%	−7.7%	− 43.8%
	CPS-TUS	22.3%	18.1%	−18.6%	14.3%	−21.1%	12.8%	−10.9%	−42.7%
	95% CI		(17.9, 18.4%)		(14.1, 14.5%)		(12.5, 13.0%)		
18–24	SimSmoke	23.6%	19.5%	−17.5%	16.3%	−16.3%	16.1%	−1.1%	−31.7%
	CPS-TUS	23.8%	23.3%	−2.2%	15.5%	−33.5%	12.1%	−22.0%	−49.2%
	95% CI		(22.3, 24.3%)		(14.7, 16.4%)		(11.3, 13.0%)		
25–34	SimSmoke	27.3%	20.9%	−23.5%	17.6%	−15.8%	16.8%	−4.6%	−38.6%
	CPS-TUS	27.6%	20.1%	−27.1%	17.2%	−14.7%	15.0%	−12.9%	−45.9%
	95% CI		(19.5, 20.8%)		(16.6, 17.8%)		(14.4, 15.6%)		
35–54	SimSmoke	25.1%	19.5%	−22.2%	14.2%	− 27.1%	12.7%	−10.7%	−49.4%
	CPS-TUS	25.1%	21.8%	−13.1%	17.2%	−20.9%	15.6%	−9.6%	−37.9%
	95% CI		(21.3, 22.2%)		(16.8, 17.6%)		(15.2, 16.0%)		
55+	SimSmoke	14.4%	12.1%	−15.6%	9.8%	−18.9%	9.1%	−7.8%	− 36.9%
	CPS-TUS	14.8%	11.4%	−22.5%	10.1%	−12.2%	9.8%	−2.2%	−33.4%
	95% CI		(11.0, 11.8%)		(9.7, 10.4%)		(9.5, 10.1%)		
Dual use									
Male									
18+	SimSmoke	1.0%	0.9%	−14.6%	0.7%	−16.4%	0.7%	−5.7%	−32.6%
	CPS-TUS	1.0%	0.5%	−47.0%	0.5%	−11.4%	0.5%	0.0%	−53.0%
	95% CI		(0.5, 0.6%)		(0.4, 0.5%)		(0.4, 0.5%)		
18–24	SimSmoke	2.2%	1.5%	−33.5%	1.3%	−8.8%	1.3%	−0.3%	−39.5%
	CPS-TUS	2.3%	1.1%	−52.0%	1.1%	0.5%	1.1%	0.0%	−51.7%
	95% CI		(0.8, 1.4%)		(0.9, 1.4%)		(0.8, 1.4%)		
25–34	SimSmoke	1.4%	1.4%	0.7%	1.0%	−31.9%	0.9%	−1.7%	−32.6%
	CPS-TUS	1.4%	1.0%	−31.0%	0.8%	−17.8%	0.9%	7.6%	−39.0%
	95% CI		(0.8, 1.1%)		(0.7, 1.0%)		(0.7, 1.0%)		
35–54	SimSmoke	0.8%	0.8%	5.8%	0.8%	−1.9%	0.7%	−8.8%	−5.4%
	CPS-TUS	0.8%	0.5%	−40.5%	0.5%	6.7%	0.5%	10.4%	−30.0%
	95% CI		(0.4, 0.5%)		(0.4, 0.6%)		(0.4, 0.6%)		
55+	SimSmoke	0.5%	0.4%	−23.9%	0.3%	−14.8%	0.3%	−0.2%	−35.3%
	CPS-TUS	0.5%	0.2%	−64.3%	0.2%	−11.1%	0.1%	−6.7%	−70.4%
	95% CI		(0.1, 0.2%)		(0.1, 0.2%)		(0.1, 0.2%)		
Female									
18+	SimSmoke	0.05%	0.03%	−33.3%	0.02%	−32.4%	0.02%	−13.2%	−60.9%
	CPS-TUS	0.05%	0.02%	−62.5%	0.01%	−44.3%	0.02%	100.0%	−58.2%
	95% CI		(0.01, 0.03%)		(0.01, 0.02%)		(0.01, 0.03%)		
18–24	SimSmoke	0.05%	0.03%	−32.2%	0.03%	−9.1%	0.03%	−0.48%	−38.7%
	CPS-TUS	0.05%	0.01%	−77.1%	0.08%	555.7%	0.02%	−75.0%	−62.5%
	95% CI		(0.00, 0.03%)		(0.04, 0.18%)		(0.00, 0.10%)		
25–34	SimSmoke	0.03%	0.03%	−2.6%	0.02%	−33.4%	0.02%	−2.8%	− 36.9%
	CPS-TUS	0.02%	0.02%	−6.3%	0.02%	−9.9%	0.01%	−50.0%	−57.8%
	95% CI		(0.00, 0.04%)		(0.01, 0.06%)		(0.00, 0.05%)		
35–54	SimSmoke	0.06%	0.03%	−49.1%	0.02%	−42.7%	0.01%	−18.6%	−76.3%
	CPS-TUS	0.06%	0.02%	−69.0%	0.01%	−43.2%	0.03%	200.0%	−47.2%
	95% CI		(0.00, 0.03%)		(0.00, 0.03%)		(0.01, 0.05%)		
55+	SimSmoke	0.05%	0.04%	−26.3%	0.02%	−34.0%	0.02%	−19.5%	−60.8%
	CPS-TUS	0.05%	0.02%	−66.6%	0.00%	−100.0%	0.01%	…	−81.0%
	95% CI		(0.00, 0.03%)		(0.00, 0.02%)		(0.00, 0.03%)		
Exclusive smokeless tobacco use									
Male									
18+	SimSmoke	3.2%	2.8%	−13.6%	2.5%	−9.3%	2.4%	−3.3%	−24.2%
	CPS-TUS	3.1%	2.3%	−27.1%	2.5%	8.0%	2.6%	6.5%	−16.1%
	95% CI		(2.1, 2.4%)		(2.3, 2.6%)		(2.5, 2.7%)		
18–24	SimSmoke	4.9%	3.6%	−26.9%	3.8%	5.9%	3.8%	0.5%	−22.2%
	CPS-TUS	5.0%	1.8%	−63.5%	2.3%	28.2%	2.9%	24.0%	−42.0%
	95% CI		(1.5, 2.2%)		(2.0, 2.8%)		(2.5, 3.4%)		
25–34	SimSmoke	4.1%	3.9%	−4.5%	3.2%	−16.9%	3.3%	3.2%	−18.1%
	CPS-TUS	4.2%	3.6%	−14.2%	3.0%	−17.4%	3.0%	3.1%	−27.0%
	95% CI		(3.2, 3.9%)		(2.7, 3.3%)		(2.8, 3.4%)		
35–54	SimSmoke	2.3%	2.5%	4.8%	2.6%	4.2%	2.4%	−5.0%	3.7%
	CPS-TUS	2.3%	2.2%	−4.1%	3.1%	42.1%	3.4%	9.4%	49.0%
	95% CI		(2.0, 2.4%)		(2.9, 3.3%)		(3.2, 3.6%)		
55+	SimSmoke	2.7%	2.0%	−25.6%	1.4%	−27.2%	1.4%	−6.0%	−49.0%
	CPS-TUS	2.7%	1.8%	−36.0%	1.6%	−9.9%	1.7%	9.5%	−36.8%
	95% CI		(1.6, 1.9%)		(1.4, 1.7%)		(1.6, 1.9%)		
Female									
18+	SimSmoke	0.4%	0.2%	−42.7%	0.1%	−39.3%	0.1%	−16.4%	−70.9%
	CPS-TUS	0.4%	0.2%	−60.4%	0.1%	−42.4%	0.1%	0.0%	−77.2%
	95% CI		(0.1, 0.2%)		(0.1, 0.1%)		(0.1, 0.1%)		
18–24	SimSmoke	0.1%	0.1%	−24.5%	0.1%	5.5%	0.1%	0.6%	−19.8%
	CPS-TUS	0.1%	0.0%	−62.4%	0.1%	86.9%	0.1%	−12.5%	−38.5%
	95% CI		(0.0, 0.1%)		(0.0, 0.2%)		(0.0, 0.2%)		
25–34	SimSmoke	0.1%	0.1%	−24.1%	0.1%	−10.4%	0.1%	2.9%	−30.0%
	CPS-TUS	0.1%	0.1%	−20.0%	0.1%	−48.8%	0.1%	66.7%	−31.7%
	95% CI		(0.1, 0.2%)		(0.0, 0.1%)		(0.1, 0.2%)		
35–54	SimSmoke	0.2%	0.1%	−44.9%	0.1%	−23.1%	0.1%	−9.7%	−61.8%
	CPS-TUS	0.2%	0.1%	−54.0%	0.1%	−8.0%	0.1%	0.0%	−57.7%
	95% CI		(0.1, 0.1%)		(0.1, 0.1%)		(0.1, 0.1%)		
55+	SimSmoke	0.9%	0.4%	−47.4%	0.2%	−54.1%	0.1%	−28.0%	−82.6%
	CPS-TUS	0.9%	0.3%	−67.9%	0.1%	−58.4%	0.1%	−16.7%	−88.8%
	95% CI		(0.2, 0.4%)		(0.1, 0.2%)		(0.1, 0.1%)		

^**a**^Relative change measured as the absolute difference in prevalence between the end and the initial year of the specified period divided by the prevalence of the initial year

For the adult population (ages 18 and above), *SimSmoke* predicted that exclusive male (female) cigarette prevalence fell from 25.6% (22.1%) in 1993 to 14.2% (12.4%) in 2015, while the TUS showed a decline from 25.7% (22.3%) to 14.9% (12.8%). The 2015 *SimSmoke* male (female) projected prevalence were outside the TUS 95% CI, but the relative reductions between 1993 and 2015 were 44.7% for males and 43.8% for females and were within 3% of the TUS estimates for both males (41.9%) and females (42.7%). By sub-periods, *SimSmoke* over-estimated the relative reduction in exclusive smoking from 1993 to 2002 less for females (− 21.4% vs. − 18.6%) than for males (− 21.3 vs. -14.1%), did better for males (− 23.5% vs − 22.5%) than females (− 22.5 vs. -21.1%) for 2002–2011, and underestimated the 2011–2013 reduction similarly for males (− 8.2% vs. -12.6%) and females (− 7.7% vs. -10.9%). In examining trends by age group, the biggest discrepancies were for 18–24 year olds, where *SimSmoke* over-predicted male and female reductions during the period 1993–2002, which was then reversed in 2002–2011 and 2011–2015.

Adult male (female) estimates from SimSmoke for dual use fell from 1.0% (0.05%) in 1993 to 0.7% (0.02%) in 2015, compared to TUS estimates of 0.05 (0.02). Compared to the TUS, the 2015 projections were within the 95% CI for females (falling from 0.5 to 0.2%), but outside the 95% CI for males. *SimSmoke* under-predicted male reductions in 1993–2002 and over-predicted the reductions in 2002–2011 and 2011–2015, but underestimated female reductions for 1993–2002 and 2002–2011 and over-predicted for 2011–2015. Similar results were obtained for most age groups.

Male (female) exclusive SLT use estimated by *Simsmoke* fell from 3.2% (0.4%) in 1993 to 2.4% (0.1%) in 2015, yielding a 24% (71%) relative reduction between 1993 and 2015 compared to a 16% (77%) relative reduction in TUS. Female projections for 2015 were marginally within the 95% CI of the TUS, while the male SLT projection was just outside the 95% CI. *SimSmoke* underestimated male relative reduction for 1993–2002 and overestimated relative reductions for 2002–2011 and 2011–2015, while female relative reductions were underestimated in first two sub-periods and then reversed in 2011–2015. Discrepancies were particularly large in the 18–24 age group.

### The effect of policies implemented through 2017

Results comparing exclusive smoking, dual use and exclusive SLT prevalence projections with policies implemented between 1993 and 2017 to a counterfactual with policies set to their 1993 levels (i.e., the absence of policy change) are shown in Table 3. Results for tobacco-attributable deaths and lives saved are shown in Table 4, with the last column showing the summation over the years 1993–2067 to obtain the lives saved over that period.

**Table 3:** Prevalence by Smoking Status (Exclusive Cigarette, Dual and Smokeless Tobacco Use) Projected by *SimSmoke* under Multiple Scenarios for Males and Females, 1993–2067

Prevalence	Type	1993	2003	2017	2037	2067	Relative Difference^a^	
							2017	2067
Male								
No policy change	Cigarette	25.6%	22.9%	20.9%	19.1%	18.6%	–	–
	Dual	1.05%	1.02%	1.02%	0.96%	0.93%	–	–
	SLT	3.19%	3.00%	2.86%	2.68%	2.60%	–	–
Actual/ status quo	Cigarette	25.6%	19.6%	13.6%	10.5%	9.6%	−34.8%	−48.3%
	Dual	1.05%	0.88%	0.68%	0.57%	0.52%	−32.5%	−43.6%
	SLT	3.19%	2.71%	2.38%	2.14%	2.03%	−16.5%	−21.9%
Price alone	Cigarette	25.6%	20.3%	15.8%	12.7%	11.7%	−24.5%	−37.1%
	Dual	1.05%	0.91%	0.78%	0.67%	0.62%	−23.1%	−33.3%
	SLT	3.19%	2.74%	2.49%	2.26%	2.15%	−12.8%	−17.4%
Smoke-free air law alone	Cigarette	25.6%	22.8%	20.0%	17.9%	17.3%	−4.0%	−7.1%
	Dual	1.05%	1.02%	0.98%	0.90%	0.87%	−3.9%	−6.4%
	SLT	3.19%	3.00%	2.86%	2.70%	2.62%	0.3%	0.8%
Media campaigns alone	Cigarette	25.6%	22.8%	20.8%	18.9%	18.4%	−0.6%	− 0.8%
	Dual	1.05%	1.02%	1.01%	0.95%	0.92%	−0.5%	− 0.7%
	SLT	3.19%	2.99%	2.85%	2.68%	2.59%	−0.2%	− 0.3%
Cessation treatment alone	Cigarette	25.6%	22.6%	20.2%	18.3%	17.8%	−3.4%	−4.2%
	Dual	1.05%	1.01%	0.98%	0.92%	0.89%	−3.0%	−3.8%
	SLT	3.19%	2.98%	2.81%	2.62%	2.54%	−1.6%	−2.3%
Health warning alone	Cigarette	25.6%	22.9%	20.9%	19.1%	18.6%	0.0%	0.0%
	Dual	1.05%	1.02%	1.02%	0.96%	0.93%	0.0%	0.0%
	SLT	3.19%	3.00%	2.82%	2.64%	2.55%	−1.1%	−1.7%
Advertising ban alone	Cigarette	25.6%	22.9%	20.8%	18.9%	18.4%	−0.5%	−0.9%
	Dual	1.05%	1.02%	1.01%	0.95%	0.92%	−0.5%	−0.8%
	SLT	3.19%	3.00%	2.84%	2.67%	2.58%	−0.4%	−0.8%
Youth access alone	Cigarette	25.6%	22.8%	20.5%	18.3%	17.7%	−2.0%	−4.9%
	Dual	1.05%	1.02%	1.00%	0.93%	0.90%	−1.2%	−3.0%
	SLT	3.19%	3.00%	2.86%	2.69%	2.60%	0.0%	0.0%
Female								
No policy change	Cigarette	22.1%	19.7%	18.4%	17.1%	16.8%	–	–
	Dual	0.05%	0.03%	0.03%	0.02%	0.02%	–	–
	SLT	0.38%	0.22%	0.12%	0.08%	0.07%	–	–
Actual/ status quo	Cigarette	22.1%	16.9%	11.9%	9.3%	8.4%	−35.2%	−49.9%
	Dual	0.05%	0.03%	0.02%	0.01%	0.01%	−32.5%	−47.0%
	SLT	0.38%	0.20%	0.10%	0.07%	0.06%	−16.4%	−20.7%
Price alone	Cigarette	22.1%	17.5%	13.9%	11.3%	10.4%	−24.6%	−38.0%
	Dual	0.05%	0.03%	0.02%	0.01%	0.01%	−21.8%	− 35.9%
	SLT	0.38%	0.21%	0.11%	0.07%	0.06%	−11.7%	−15.8%
Smoke-free air law alone	Cigarette	22.1%	19.6%	17.7%	16.0%	15.6%	−4.1%	−7.4%
	Dual	0.05%	0.03%	0.02%	0.02%	0.02%	−3.9%	−6.8%
	SLT	0.38%	0.22%	0.12%	0.08%	0.07%	0.2%	1.1%
Media campaign alone	Cigarette	22.1%	19.6%	18.3%	17.0%	16.7%	−0.6%	−0.8%
	Dual	0.05%	0.03%	0.03%	0.02%	0.02%	−0.5%	−0.7%
	SLT	0.38%	0.22%	0.12%	0.08%	0.07%	−0.2%	−0.3%
Cessation treatment alone	Cigarette	22.1%	19.5%	17.7%	16.3%	16.0%	−3.8%	−5.2%
	Dual	0.05%	0.03%	0.02%	0.02%	0.02%	−4.3%	−4.6%
	SLT	0.38%	0.22%	0.12%	0.08%	0.07%	−2.5%	−2.8%
Health warning alone	Cigarette	22.1%	19.7%	18.4%	17.1%	16.8%	0.0%	0.0%
	Dual	0.05%	0.03%	0.03%	0.02%	0.02%	0.0%	0.0%
	SLT	0.38%	0.22%	0.12%	0.08%	0.07%	−1.2%	−1.9%
Advertising ban alone	Cigarette	22.1%	19.7%	18.3%	17.0%	16.7%	−0.5%	−0.9%
	Dual	0.05%	0.03%	0.03%	0.02%	0.02%	−0.5%	−0.8%
	SLT	0.38%	0.22%	0.12%	0.08%	0.07%	−0.4%	−0.8%
Youth access alone	Cigarette	22.1%	19.7%	18.1%	16.5%	16.0%	−1.9%	−4.7%
	Dual	0.05%	0.03%	0.03%	0.02%	0.02%	−1.1%	−3.8%
	SLT	0.38%	0.22%	0.12%	0.08%	0.07%	0.0%	0.3%

^a^Relative differences measured as the absolute difference between current prevalence and no-policy-change scenario prevalence from the same year divided by the no-policy-change prevalence for the same year

**Table 4:** Tobacco-Attributable Deaths by Smoking Status Projected by SimSmoke under Multiple Scenarios for Males and Females, 1993–2067

Policies	Type	1993	2003	2017	2037	2067	Cumulative	
							1993–2017	1993–2067
Male								
Tobacco-Attributable Deaths with Policies								
Actual/ status quo	Cigarette	214,536	235,471	238,852	200,634	144,977	5,850,036	15,175,074
	Dual	7072	6755	7085	8195	6859	172,098	550,611
	SLT	5371	5898	5243	5368	5321	141,452	406,886
	Total	226,979	248,123	251,180	214,196	157,158	6,163,585	16,132,572
Lives Saved Compared to the Counterfactual of No Policy Change^a^								
Actual/ status quo	Cigarette	–	4167	33,407	71,464	128,514	257,655	4,350,888
	Dual	–	126	1011	3141	5747	7520	186,594
	SLT	–	68	381	949	1700	3453	57,979
	Total	–	4362	34,800	75,553	135,961	268,628	4,595,461
Price alone	Cigarette	–	3058	20,160	45,179	96,264	162,609	2,959,865
	Dual	–	92	600	1962	4278	4644	126,743
	SLT	–	60	282	659	1288	2759	42,282
	Total	–	3210	21,042	47,801	101,831	170,013	3,128,890
Smoke-free air law alone	Cigarette	–	48	2288	9212	18,973	12,291	549,538
	Dual	–	0	0	0	0	0	0
	SLT	–	1	74	412	843	386	24,265
	Total	–	49	2362	9623	19,816	12,677	573,445
Media campaign alone	Cigarette	–	42	573	1182	2252	4033	75,506
	Dual	–	1	18	52	98	118	3234
	SLT	–	0	3	10	22	21	667
	Total	–	43	594	1245	2373	4172	79,406
Cessation treatment alone	Cigarette	–	328	5801	13,195	16,515	38,207	693,120
	Dual	–	9	174	596	773	1098	30,665
	SLT	–	2	50	173	266	308	9589
	Total	–	339	6025	13,963	17,554	39,613	733,373
Health warning alone	Cigarette	–	0	0	0	0	0	0
	Dual	–	0	0	0	0	0	6
	SLT	–	0	17	73	134	85	4166
	Total	–	0	17	73	134	85	3596
Advertising ban alone	Cigarette	–	0	319	1069	2123	1205	62,167
	Dual	–	0	11	47	93	42	2703
	SLT	–	0	5	21	47	27	1296
	Total	–	0	335	1137	2263	1274	66,165
\Youth access alone	Cigarette	–	0	17	1824	10,683	26	189,846
	Dual	–	0	1	63	348	1	6343
	SLT	–	0	0	1	1	0	44
	Total	–	0	18	1887	11,032	27	196,232
Female								
Tobacco-Attributable Deaths with Policies								
Actual/ status quo	Cigarette	125,607	140,968	146,742	142,518	102,473	3,508,793	9,961,334
	Dual	506	443	364	221	103	10,857	20,938
	SLT	2078	1863	969	353	173	41,759	60,235
	Total	128,191	143,275	148,076	143,093	102,749	3,561,410	10,042,508
Lives Saved Compared to the Counterfactual of No Policy Change^a^								
Actual/ status quo	Cigarette	–	2617	21,559	48,462	90,074	165,934	2,932,063
	Dual	–	10	48	63	91	436	3845
	SLT	–	27	72	49	46	938	3484
	Total	–	2653	21,679	48,574	90,212	167,308	2,939,392
Price alone	Cigarette	–	1936	13,098	29,304	67,050	106,278	1,954,695
	Dual	–	7	28	36	68	275	2462
	SLT	–	23	51	32	34	751	2504
	Total	–	1967	13,178	29,372	67,152	107,304	1,959,661
Smoke-free air law alone	Cigarette	–	28	1393	6274	13,254	7360	369,202
	Dual	–	0	0	0	0	0	0
	SLT	–	0	3	8	13	18	451
	Total	–	29	1396	6282	13,267	7378	369,639
Media campaign alone	Cigarette	–	24	353	798	1654	2434	51,313
	Dual	–	0	1	1	2	6	65
	SLT	–	0	1	1	1	5	33
	Total	–	24	354	800	1656	2445	51,411
Cessation treatment alone	Cigarette	–	201	3676	9978	12,899	23,840	513,312
	Dual	–	1	9	14	13	62	717
	SLT	–	1	10	10	8	81	572
	Total	–	202	3695	10,003	12,920	23,983	514,601
Health warning alone	Cigarette	–	0	0	0	0	0	0
	Dual	–	0	0	0	0	0	0
	SLT	–	0	4	4	4	23	218
	Total	–	0	4	4	4	23	211
Advertising ban alone	Cigarette	–	0	195	735	1472	724	41,862
	Dual	–	0	0	1	1	2	51
	SLT	–	0	1	1	1	7	64
	Total	–	0	197	738	1475	734	41,977
Youth access alone	Cigarette	–	0	7	822	6598	11	103,780
	Dual	–	0	0	1	6	0	100
	SLT	–	0	0	0	0	0	0
	Total	–	0	7	823	6604	11	103,880

^a^ Lives saved were calculated as the difference in projected deaths with the policy implemented and with no policy implemented

In 1993, total tobacco-attributable deaths for males (females) were estimated as 226,979 (128,191), including 214,536 (125,607) exclusive smokers, 7072 (506) dual users and 5371 (2078) exclusive SLT users. For 2017, *SimSmoke* projected 251,180 (148,076) total attributable deaths, including 238,852 (146,076) exclusive smokers, 7085 (364) dual users and 5243 (969) exclusive SLT users. Since 1993, premature deaths generally grew and then declined in number, except among female dual and exclusive SLT users which showed continuous decline.

With no new policies implemented after 1993, *SimSmoke* projected that exclusive cigarette, dual and exclusive SLT use rates would have been 35, 32.5 and 16.5% higher respectively in 2017 for males, with similar relative differences for females. As a result of policies, annual tobacco-attributable deaths for males (females) were reduced by 34,800 (21,679) in 2017 alone with a cumulative impact of 268,628 (167,308) fewer tobacco-attributable deaths from 1993 to 2017. By 2067, the relative reductions for males (females) increased to 48% (50%) for exclusive cigarette, 44% (47%) for dual and 22% (21%) for exclusive SLT users, as policies continued to reduce tobacco use through increased cessation and reduced initiation. Due to policies implemented between 1993 and 2017, *SimSmoke* projected a total of 4,595,461 (2,939,392) premature deaths averted by 2067.

Comparing the counterfactual for individual policies, much of the reduction in exclusive cigarette use was due to price increases. Price increases alone were predicted to reduce male (female) exclusive cigarette use rates in relative terms by 25% (25%) in 2017 and by 37% (38%) in 2067, and to have averted 3,128,890 (1,959,661) male (female) deaths in total by 2067. Smoke-free air laws yielded a 4% relative reduction in exclusive cigarette use in 2017, which increased to a 7% reduction by 2067. Cessation treatments and youth access enforcement showed 3–4 and 2% relative reductions respectively in 2017 increasing to 4–5 and 5% by 2067. Mass media campaigns and advertising bans showed 0.6 and 0.5% relative reductions respectively in 2017 increasing to 0.8 and 0.9% reductions by 2067. For exclusive cigarettes, taxes represented 71% of the total policy effects, followed by smoke-free air laws at 11%, and cessation treatment at 10% by 2017.

Similar but slightly smaller relative reductions were projected for dual use. However, much smaller effects were projected for exclusive SLT use, where the largest relative reductions by 2067 for males (females) were 13% (12%) for prices, followed by 1.6% (2.5%) for cessation treatment and 1.1% (1.2%) for health warnings. Some categories show increased exclusive SLT use in future years, due to the larger pool of potential initiates from those who would have smoked cigarettes.

## Discussion

Our estimates of the increase in exclusive cigarette use between 1993 and 2015 from US *SimSmoke* generally validated well against trends found in the large scale, nationally representative TUS. However, *SimSmoke* over-estimated reductions among male smokers for most ages, especially those 18–24, until 2002, while under-estimating reductions in later years. By 2015, *SimSmoke* female projections of adult exclusive and dual cigarette use were close to TUS estimates, while male reductions were under-estimated for dual use but over-estimated for exclusive SLT use. The deviations for dual use may reflect the relatively small number of such users. Contrary to the results for exclusive cigarette use, both male exclusive SLT use and male dual use underestimated the reductions for 1993–2002, while moving closer to the TUS estimates by 2015. These reversals were particularly apparent for the 18–24 and 35–54 age groups.

Consistent with previous literature [8, 9], the model projected that overall SLT rates fell quite rapidly for both dual and exclusive SLT use through 2002, but decelerated in recent years. However, *SimSmoke* under-predicted the decline through 2002. While some policies were directed at SLT use between 1993 and 2002, most were directed at cigarette use, including tax increases, smoke-free air laws, and media campaigns. These policies may have also reduced SLT use, suggesting the importance of strong cigarette policies in reducing overall tobacco use.

The model fails to predict well the increasing pattern of exclusive SLT and dual use found in recent TUS surveys and in recent studies [6, 10, 11, 82, 83]. The failure to predict these changes in trend may reflect the changing composition of the SLT industry. Reynolds American acquired Conwood Smokeless Tobacco Company in 2006 and soon thereafter introduced Camel Snus, and Altria acquired the U.S. Smokeless Tobacco Company in 2009 and began marketing Marlboro Snus. Together they controlled 85% of the market [13]. Industry documents [84, 85] indicate that cigarette companies began promoting SLT products as a way for smokers to satisfy nicotine cravings in places where smoking is banned, and marketing expenditures, including those on price promotions [86] and flavored products [87, 88], increased. The largest increases in SLT use were among young adults, possibly reflecting marketing targeted toward this age group. Policies may need to be directed at this age group in order to reduce SLT and dual use.

*SimSmoke* projected that policies implemented between 1993 and 2017 reduced cigarette use by about 35% and SLT use by 16.5%. Consistent with earlier *SimSmoke* analyses [89, 90], the largest percentage reductions in cigarette and SLT use and in attributable deaths were due to taxes. Smoke-free air laws were next most important for cigarettes, while cessation treatment was next most important for SLTs. The importance of taxes and smoke-free air laws has also been found in previous US *SimSmoke* models of cigarette use [2, 20–22, 25, 26].

*SimSmoke* also provided estimates of the health effects of SLT use. *SimSmoke* estimated 6212 deaths attributable to exclusive SLT use in 2017 (down from 7449 in 1993), but projected general increases in future years. However, we treated SLT as a homogeneous category in terms of risks, potentially overestimating risks (e.g., SLT users switching to snus) [91–95]. The number of SLT-attributable deaths paled in comparison to the total deaths attributable to dual and exclusive cigarette use, which were estimated as 7449 and 385,594 respectively in 2017. The model did not distinguish the relative risks of dual use from that of exclusive cigarette use, although dual use may reduce the number of cigarettes smoked over a lifetime and, thereby, reduce mortality risks.

Like all models, *SimSmoke* estimates are only as strong as the assumptions and underlying data. In particular, the projections of cigarette use were based on initiation and cessation rates derived in 1993 subject to policy changes over time. Cessation rates for exclusive SLT users were not available, and we were not able to distinguish cessation rates for dual as compared to exclusive cigarette use. In addition, the effect sizes of policies on SLT use that we used in *SimSmoke*, are tentative, largely reflecting studies prior to 2007 [17]. Better information is needed on policy effectiveness, especially for recent years since the cigarette companies came to dominate the industry, and on the extent to which policies, such as media campaigns, are directed at SLT use. Better information is also needed about the timing of policies effects and the potential synergies or overlapping effects of policies as they relate to cigarette and SLT use.

Another limitation is that *SimSmoke* considers only cigarette and SLT use, and does not include the use of other nicotine delivery products, such as cigars, water pipes and e-cigarettes, that may substitute or complement the use of cigarettes and SLT. Growth in e-cigarette use between 2011 and 2015 [96, 97] may explain the rapid reduction in cigarette use and the slowing growth of SLT use.

## Conclusions

While the landscape for nicotine delivery products has dramatically changed in the last 10 years, some lessons can be gleaned from the modeling in this paper. With cigarettes still being the dominant form of nicotine delivery, cigarette-oriented policies may be an effective means, perhaps the most effective means, of reducing SLT use and possibly reducing the use of other nicotine delivery products, such as e-cigarettes. Policies directed at SLT use, especially those that affect youth and young adults, may also play a role but it should be recognized that substitution of exclusive SLT use (which is relatively low risk) for cigarette use can reduce overall harms. In developing a coherent policy approach, it will be important to monitor the use of other products, such as cigars and e-cigarettes. In addition, it will be important to monitor the marketing and pricing policies of cigarette companies, which have strong incentives to protect the high profit margins of cigarettes.

## Abbreviations

FSPTCA: Smoking Prevention and Tobacco Control Act
SLT: smokeless tobacco

## References

[1] The 2014-2015 Tobacco Use Supplement to the Current Population Survey (TUS-CPS). [https://cancercontrol.cancer.gov/brp/tcrb/tus-cps/TUS-CPS_2014-15_SummaryDocument.pdf].

[2] Levy DT, Meza R, Zhang Y, Holford TR (2016). Gauging the effect of U.S. tobacco control policies from 1965 through 2014 using SimSmoke. Am J Prev Med.

[3] Levy DT, Nikolayev N, Mumford EA (2005). Recent trends in smoking and the role of public policies: results from the SimSmoke tobacco control policy simulation model. Addiction.

[4] Lee YO, Hebert CJ, Nonnemaker JM, Kim AE (2014). Multiple tobacco product use among adults in the United States: cigarettes, cigars, electronic cigarettes, hookah, smokeless tobacco, and snus. Prev Med.

[5] Sung HY, Wang Y, Yao T, Lightwood J, Max W (2016). Polytobacco use of cigarettes, cigars, chewing tobacco, and snuff among US adults. Nicotine Tob Res.

[6] U.S. Department of Health and Human Services (2014). The Health Consequences of Smoking—50 Years of Progress: A Report of Surgeon General.

[7] Kasza KA, Ambrose BK, Conway KP, Borek N, Taylor K, Goniewicz ML, Cummings KM, Sharma E, Pearson JL, Green VR (2017). Tobacco-product use by adults and youths in the United States in 2013 and 2014. N Engl J Med.

[8] Nelson DE, Mowery P, Tomar S, Marcus S, Giovino G, Zhao L (2006). Trends in smokeless tobacco use among adults and adolescents in the United States. Am J Public Health.

[9] Mumford EA, Levy DT, Gitchell JG, Blackman KO (2005). Tobacco control policies and the concurrent use of smokeless tobacco and cigarettes among men, 1992-2002. Nicotine Tob Res.

[10] Agaku IT, Alpert HR (2016). Trends in annual sales and current use of cigarettes, cigars, roll-your-own tobacco, pipes, and smokeless tobacco among US adults, 2002-2012. Tob Control.

[11] Chang JT, Levy DT, Meza R (2016). Trends and factors related to smokeless tobacco use in the United States. Nicotine Tob Res.

[12] Sales of Tobacco Products. 2015. [https://www.ttb.gov/statistics/16tobstats.shtml].

[13] National Cancer Institute and Centers for Disease Control and Prevention (2014). Report on smokeless tobacco and public health: a global perspective.

[14] Freiberg M, Boyle RG, Moilanen M, St Claire AW, Weisman SR (2014). The land of 10,000 tobacco products: how Minnesota led the way in regulating tobacco products. Am J Public Health.

[15] Levy DT, Mays D, Boyle RG, Tam J, Chaloupka FJ (2017). The effect of tobacco control policies on US smokeless tobacco use: a structured review. Nicotine Tob Res.

[16] Levy DT, Cummings KM, Villanti AC, Niaura R, Abrams DB, Fong GT, Borland R (2017). A framework for evaluating the public health impact of e-cigarettes and other vaporized nicotine products. Addiction.

[17] Levy DT, Bauer JE, Lee HR (2006). Simulation modeling and tobacco control: creating more robust public health policies. Am J Public Health.

[18] Levy D, de Almeida LM, Szklo A (2012). The Brazil SimSmoke policy simulation model: the effect of strong tobacco control policies on smoking prevalence and smoking-attributable deaths in a middle income nation. PLoS Med.

[19] Levy D, Rodriguez-Buno RL, Hu TW, Moran AE (2014). The potential effects of tobacco control in China: projections from the China SimSmoke simulation model. BMJ.

[20] Levy D, Tworek C, Hahn E, Davis R (2008). The Kentucky SimSmoke tobacco policy simulation model: reaching healthy people 2010 goals through policy change. South Med J.

[21] Levy DT, Benjakul S, Ross H, Ritthiphakdee B (2008). The role of tobacco control policies in reducing smoking and deaths in a middle income nation: results from the Thailand SimSmoke simulation model. Tob Control.

[22] Levy DT, Boyle RG, Abrams DB (2012). The role of public policies in reducing smoking: the Minnesota SimSmoke tobacco policy model. Am J Prev Med.

[23] Levy DT, Huang AT, Currie LM, Clancy L (2014). The benefits from complying with the framework convention on tobacco control: a SimSmoke analysis of 15 European nations. Health Policy Plan.

[24] Levy DT, Huang AT, Havumaki JS, Meza R (2016). The role of public policies in reducing smoking prevalence: results from the Michigan SimSmoke tobacco policy simulation model. Cancer Causes Control.

[25] Levy DT, Hyland A, Higbee C, Remer L, Compton C (2007). The role of public policies in reducing smoking prevalence in California: results from the California tobacco policy simulation model. Health Policy.

[26] Levy DT, Ross H, Powell L, Bauer JE, Lee HR (2007). The role of public policies in reducing smoking prevalence and deaths caused by smoking in Arizona: results from the Arizona tobacco policy simulation model. J Public Health Manag Pract.

[27] Population Estimates 1993-1999 [http://www.census.gov/population/estimates/nation/infile2-1.test].

[28] Population Estimates 2010-2015 [http://factfinder.census.gov/faces/tableservices/jsf/pages/productview.xhtml?src=bkmk].

[29] Population Estimates 2000-2009 [http://www.census.gov/popest/data/intercensal/state/files/ST-EST00INT-AGESEX.csv].

[30] Population Projections [https://www2.census.gov/programs-surveys/popproj/datasets/2014/2014-popproj/np2014_d1.csv].

[31] Fertility rates, mortality and birth rates by age and gender [http://wonder.cdc.gov/cmf-icd10.html].

[32] Immigration Rates [https://www2.census.gov/programs-surveys/popproj/datasets/2014/2014-popproj/np2014_d4.csv].

[33] U.S. Bureau of the Census. Current Population Survey, September 1993: Tobacco Use Supplement File, Technical Documentation CPS-01. Washington, DC: National Cancer Institute, National Institute of Health; 2001.

[34] Hatsukami DK, Henningfield JE, Kotlyar M (2004). Harm reduction approaches to reducing tobacco-related mortality. Annu Rev Public Health.

[35] Hatsukami DK, Lemmonds C, Tomar SL (2004). Smokeless tobacco use: harm reduction or induction approach?. Prev Med.

[36] Phillips CV, Heavner KK (2009). Smokeless tobacco: the epidemiology and politics of harm. Biomarkers.

[37] Tomar SL (2003). Is use of smokeless tobacco a risk factor for cigarette smoking? The U.S. experience. Nicotine Tob Res.

[38] Tomar SL (2007). Epidemiologic perspectives on smokeless tobacco marketing and population harm. Am J Prev Med.

[39] Burns DM, Anderson C, Major J, Vaughn J, Shanks T (2000). Cessation and cessation measures among daily adult smokers: national- and state-specific data. *Population Based Smoking Cessation Monograph No 12.* Edn. Washington, DC: National Institutes of Health. National Cancer Institute. NIH publication no. 00–4804, 2000.39.

[40] Zhu SH, Wang JB, Hartman A, Zhuang Y, Gamst A, Gibson JT, Gilljam H, Galanti MR (2009). Quitting cigarettes completely or switching to smokeless tobacco: do US data replicate the Swedish results?. Tob Control.

[41] Tam J, Day HR, Rostron BL, Apelberg BJ (2015). A systematic review of transitions between cigarette and smokeless tobacco product use in the United States. BMC Public Health.

[42] Chang J, Levy DT, Meza R. Examining the transitions between cigarette and smokeless tobacco product use in the United States using the 2002-2003 and 2010-2011 longitudinal cohorts. Nicotine & Tob Res. 2017. In press. 10.1093/ntr/ntx251 PMC6154991 29126271

[43] Frost-Pineda K, Appleton S, Fisher M, Fox K, Gaworski CL (2010). Does dual use jeopardize the potential role of smokeless tobacco in harm reduction?. Nicotine Tob Res.

[44] Schauer GL, Pederson LL, Malarcher AM (2016). Past year quit attempts and use of cessation resources among cigarette-only smokers and cigarette smokers who use other tobacco products. Nicotine Tob Res.

[45] Messer K, Vijayaraghavan M, White MM, Shi Y, Chang C, Conway KP, Hartman A, Schroeder MJ, Compton WM, Pierce JP (2015). Cigarette smoking cessation attempts among current US smokers who also use smokeless tobacco. Addict Behav.

[46] McWhorter WP, Boyd GM, Mattson ME (1990). Predictors of quitting smoking: the NHANES I followup experience. J Clin Epidemiol.

[47] Gilpin EA, Pierce JP, Farkas AJ (1997). Duration of smoking abstinence and success in quitting. J Natl Cancer Inst.

[48] U.S. DHHS (1989). Reducing the health consequences of smoking: 25 years of progress: a report of Surgeon General.

[49] U.S. DHHS (1990). The Health Benefits of Smoking Cessation: a report of Surgeon General.

[50] Thun MJ, Myers DG, Day-Lally C, Namboodiri NM, Calle EE, Flanders WD, Adams SL, CWJ H, Institute NC (1997). Age and the exposure-response relationships between cigarette smoking and premature death in Cancer Prevention Study II. Changes in cigarette related disease risks and their implication for prevention and control.

[51] Burns D, Garfinkel L, Samet J (1997). Changes in cigarette-related disease risks and their implication for prevention and control.

[52] Krautter GR, Borgerding MF (2014). Comparison of consumption patterns, biomarkers of exposure, and subjective effects in cigarette smokers who switched to dissolvable tobacco (camel orbs), dual use, or tobacco abstinence. Nicotine Tob Res.

[53] Lee PN (2014). Health risks related to dual use of cigarettes and snus - a systematic review. Regul Toxicol Pharmacol.

[54] Krautter GR, Chen PX, Borgerding MF (2015). Consumption patterns and biomarkers of exposure in cigarette smokers switched to snus, various dissolvable tobacco products, dual use, or tobacco abstinence. Regul Toxicol Pharmacol.

[55] Henley SJ, Thun MJ, Connell C, Calle EE (2005). Two large prospective studies of mortality among men who use snuff or chewing tobacco (United States). Cancer Causes Control.

[56] Levy DT, Cummings KM, Hyland A (2000). Increasing taxes as a strategy to reduce cigarette use and deaths: results of a simulation model. Prev Med.

[57] Levy DT, Tam J, Kuo C, Fong GT, Chaloupka F. The impact of implementing tobacco control policies: the 2017 tobacco control policy scorecard. J Public Health Manag Pract. 2018. [Epub ahead of print]. 10.1097/PHH.0000000000000780 PMC6050159 29346189

[58] Orzechowski, Walker. The tax burden on tobacco, historical compilation. Arlington: Orzechowski and Walker; 2014.

[59] Levy DT, Yuan Z, University G (2017). Prices and Taxes of Smokeless Tobacco.

[60] Smokeless Report For 2014 (Issued 2016). Chicago: Univeristy of Illinois.

[61] Huang J, Chaloupka F (2011). The economic impact of state cigarette taxes and smoke-free air policies on convenience stores. Research Paper Series.

[62] Levy DT, Friend K, Polishchuk E (2001). Effect of clean indoor air laws on smokers: the clean air module of the SimSmoke computer simulation model. Tob Control.

[63] Summary of 100% Smokefree State Laws and Population Protected by 100% U.S. Smokefree Laws [www.non-smokersrights.org].

[64] Huang J, Walton K, Gerzoff RB, King BA, Chaloupka FJ (2015). Centers for disease C, prevention: state tobacco control program spending--United States, 2011. MMWR Morb Mortal Wkly Rep.

[65] Levy DT, Friend K (2001). A computer simulation model of mass media interventions directed at tobacco use. PrevMed.

[66] Factsheet: State Tob Control Expenditures, 2000-2016 [http://www.tobaccofreekids.org/research/factsheets/pdf/0209.pdf].

[67] Levy DT, Chaloupka F, Gitchell J (2004). The effects of tobacco control policies on smoking rates: a tobacco control scorecard. Journal of public health management and practice : JPHMP.

[68] Levy DT, Lindblom EN, Fleischer NL, Thrasher J, Mohlman MK, Zhang Y, Monshouwer K, Nagelhout GE (2015). Public health effects of restricting retail tobacco product displays and ads. Tob Regul Sci.

[69] Levy DT, Mays D, Yuan Z, Hammond D, Thrasher JF (2017). Public health benefits from pictorial health warnings on US cigarette packs: a SimSmoke simulation. Tob Control.

[70] Levy D, Graham A, Mabry P, Abrams D, CT O (2010). Modeling the impact of smoking cessation treatment policies on quit rates. Am J Prev Med.

[71] Ebbert J, Montori VM, Erwin PJ, Stead LF (2011). Interventions for smokeless tobacco use cessation. Cochrane Database Syst Rev.

[72] West R, Raw M, McNeill A, Stead L, Aveyard P, Bitton J, Stapleton J, McRobbie H, Pokhrel S, Lester-George A (2015). Health-care interventions to promote and assist tobacco cessation: a review of efficacy, effectiveness and affordability for use in national guideline development. Addiction.

[73] Quitline Stats [http://www.naquitline.org/?page=800QUITNOWstats].

[74] Levy DT, Graham AL, Mabry PL, Abrams DB, Orleans CT (2010). Modeling the impact of smoking-cessation treatment policies on quit rates. Am J Prev Med.

[75] Levy DT, Friend K, Holder H, Al E (2001). Effect of policies directed at youth access to smoking: results from the SimSmoke computer simulation model. Tob Control.

[76] Chaloupka FJ, Tauras JA, Grossman M (1997). Public policy and youth smokeless tobacco use. South Econ J.

[77] Tauras J, Powell L, Chaloupka F, Ross H (2007). The demand for smokeless tobacco among male high school students in the United States: the impact of taxes, prices and policies. Appl Econ.

[78] Clark PI, Natanblut SL, Schmitt CL, Wolters C, Iachan R (2000). Factors associated with tobacco sales to minors: lessons learned from the FDA compliance checks. JAMA : the journal of the American Medical Association.

[79] Choi K, Fabian LE, Brock B, Engman KH, Jansen J, Forster JL (2014). Availability of snus and its sale to minors in a large Minnesota city. Tob Control.

[80] State Tobacco Activites Tracking and Evaluation System [http://www.cdc.gov/statesytem/].

[81] Mumford EA, Levy DT, Gitchell JG, Blackman KO (2006). Smokeless tobacco use 1992-2002: trends and measurement in the current population survey-tobacco use supplements. Tob Control.

[82] Bhattacharyya N (2012). Trends in the use of smokeless tobacco in United States, 2000-2010. Laryngoscope.

[83] Delnevo CD, Wackowski OA, Giovenco DP, Manderski MT, Hrywna M, Ling PM (2014). Examining market trends in the United States smokeless tobacco use: 2005-2011. Tob Control.

[84] Mejia AB, Ling PM (2010). Tobacco industry consumer research on smokeless tobacco users and product development. Am J Public Health.

[85] Carpenter CM, Connolly GN, Ayo-Yusuf OA, Wayne GF (2009). Developing smokeless tobacco products for smokers: an examination of tobacco industry documents. Tob Control.

[86] Richardson A, Ganz O, Stalgaitis C, Abrams D, Vallone D (2014). Noncombustible tobacco product advertising: how companies are selling the new face of tobacco. Nicotine Tob Res.

[87] Villanti AC, Richardson A, Vallone DM, Rath JM (2013). Flavored tobacco product use among U.S. young adults. Am J Prev Med.

[88] Oliver AJ, Jensen JA, Vogel RI, Anderson AJ, Hatsukami DK (2013). Flavored and nonflavored smokeless tobacco products: rate, pattern of use, and effects. Nicotine Tob Res.

[89] Levy DT, Nikolayev L, Mumford E (2005). Recent trends in smoking and the role of public policies: results from the SimSmoke tobacco control policy simulation model. Addiction.

[90] Levy DT, Nikolayev L, Mumford E, Compton C (2005). The healthy people 2010 smoking prevalence and tobacco control objectives: results from the SimSmoke tobacco control policy simulation model (United States). Cancer causes & control : CCC.

[91] Lee PN, Hamling J (2009). Systematic review of the relation between smokeless tobacco and cancer in Europe and North America. BMC Med.

[92] Richter P, Hodge K, Stanfill S, Zhang L, Watson C (2008). Surveillance of moist snuff: total nicotine, moisture, pH, un-ionized nicotine, and tobacco-specific nitrosamines. Nicotine Tob Res.

[93] Rodu B, Jansson C (2004). Smokeless tobacco and oral cancer: a review of the risks and determinants. Crit Rev Oral Biol Med.

[94] Stepanov I, Biener L, Yershova K, Nyman AL, Bliss R, Parascandola M, Hatsukami DK (2014). Monitoring tobacco-specific N-nitrosamines and nicotine in novel smokeless tobacco products: findings from round II of the new product watch. Nicotine Tob Res.

[95] National Cancer Instute and Centers for Disease Control and Prevention (2014). Report on smokeless tobacco and public health: a global perspective.

[96] Levy DT, Yuan Z, Li Y. The prevalence and characteristics of E-cigarette users in the U.S. Int J Environ Res Public Health. 2017;14(10):E1200. 10.3390/ijerph14101200 PMC5664701 29019917

[97] Marynak KL, Gammon DG, King BA, Loomis BR, Fulmer EB, Wang TW, Rogers T (2017). National and state trends in sales of cigarettes and E-cigarettes, U.S., 2011-2015. Am J Prev Med.

[98] Chaloupka FJ, Straif K, Leon ME (2011). Working group International Agency for Research on Cancer: effectiveness of tax and price policies in tobacco control. Tob Control.

[99] Jha P, Chaloupka FJ, Moore J, Gajalakshmi V, Gupta PC, Peck R, Asma S, Zatonski W. In: Jamison DT, Breman JG, Measham AR, Alleyne G, Claeson M, Evans DB, Jha P, Mills A, Musgrove P, editors. Tobacco Addiction, Disease Control Priorities in Developing Countries. 2nd EDN. Washington (DC): The International Bank for Reconstruction and Development, The World Bank; 2006. 21250309

